# Mpox Beyond Emergence: Scientific Advances, Persistent Inequities, and Future Directions

**DOI:** 10.3390/v18080808

**Published:** 2026-07-23

**Authors:** Patrick M. Katoto, Robert Colebunders, David A. Schwartz

**Affiliations:** 1 Centre for Tropical Diseases and Global Health, Catholic University of Bukavu, Bukavu 285, Democratic Republic of the Congo; 2 Centre for Equitable Immunisation and Pandemic Preparedness, Cape Town 7505, South Africa; 3 Global Health Institute, University of Antwerp, 2610 Antwerp, Belgium; robert.colebunders@uantwerpen.be; 4 Perinatal Pathology Consulting, Atlanta, GA 30342, USA; davidalanschwartz@gmail.com

The global epidemiology of mpox has undergone a profound transformation over the past decade, evolving from a historically neglected zoonotic infection into a complex and expanding international public health challenge. This Special Issue, “Mpox (Monkeypox): From Neglected Tropical Disease to Emerging Global Pathogen”, assembled nineteen multidisciplinary contributions spanning epidemiology, virology, genomics, immunology, therapeutics, maternal–fetal medicine, clinical sciences, vaccination, and global health policy. Collectively, these manuscripts document the widening geographic distribution of monkeypox virus (MPXV) lineages, the escalating burden in endemic African settings, and the emergence of novel clade I variants with heightened pathogenicity. Particular attention is devoted to the devastating impact of mpox on pregnant women and their offspring, critically understudied populations facing disproportionate risk of fetal loss, congenital infection, and maternal morbidity. The Special Issue also examines progress and shortfalls in countermeasures—antivirals, vaccines, and diagnostics—and highlights the enduring role of community engagement in outbreak response. While the science of mpox has advanced considerably, these contributions collectively expose persistent and deeply concerning disparities in genomic surveillance, implementation science, clinical trial inclusivity, countermeasure access, and international outbreak governance. This editorial synthesis reviews the principal contributions of the Special Issue, integrates emerging conceptual shifts, and identifies priority directions for the next phase of globally equitable mpox research and preparedness.

## 1. Introduction

For most of the half-century since its initial characterization in humans, mpox occupied an uneasy position within the global infectious disease landscape: scientifically recognized yet operationally neglected. Although human infection with monkeypox virus (MPXV) was first documented in 1970 in the Democratic Republic of the Congo (DRC) [1], subsequent outbreaks in Central and West Africa were routinely framed as geographically constrained zoonotic spillover events of limited international consequence. The progressive erosion of orthopoxvirus immunity following the cessation of routine smallpox vaccination passed largely unaddressed in terms of policy and preparedness.

The multicountry mpox outbreak of 2022 dismantled this complacency. The rapid international spread of clade IIb MPXV demonstrated that sustained human-to-human transmission could occur efficiently through dense sexual and social networks, particularly among gay, bisexual, and other men who have sex with men (GBMSM), affecting approximately 87,000 persons across 110 countries [2]. Simultaneously, endemic African countries continued experiencing escalating outbreaks marked by increasing case numbers, geographic expansion, and shifting demographic profiles. These parallel developments collectively invalidated the longstanding assumption that mpox represented a rare, self-limiting tropical infection of peripheral global significance.

The subsequent emergence of novel clade Ib variants—particularly in eastern DRC and South Kivu Province—introduced additional epidemiological and biological complexity. These outbreaks have demonstrated increasing adaptation to human-to-human transmission, including probable sexual amplification, urban dissemination, and disproportionately severe outcomes among pregnant women. A prospective cohort study analyzing pooled data from three cohort studies in the DRC (Uvira mpox, PREGMPOX, and MBOTE-SK) and a randomized controlled trial (PALM007) demonstrated fetal loss in 45% of women [3,4]. The possibility that distinct MPXV clades differ substantially in virulence, transmissibility, and reproductive consequences [5] has fundamentally reshaped scientific understanding of the orthopoxvirus threat landscape.

It was within this rapidly evolving context that this Special Issue was conceived and assembled. The objective was not merely to compile outbreak reports but to provide a multidisciplinary scientific platform capable of documenting and interpreting the transition of mpox from a neglected tropical disease into an emerging global pathogen with implications extending across virology, immunology, maternal–fetal health, therapeutics, community health, implementation science, and preparedness policy.

The nineteen manuscripts published in this issue collectively offer a detailed and timely portrait of the current mpox landscape. Importantly, they also show the widening gap between scientific progress and equitable implementation—a recurring and sobering theme that demands confrontation. This editorial synthesizes the principal contributions of the Special Issue, integrates the major conceptual shifts now occurring within the field, and articulates the unresolved priorities that must shape the next phase of mpox research and global preparedness.

## 2. Scientific Contributions of This Special Issue

A defining strength of this Special Issue is its multidisciplinary breadth. The collection integrates original research articles, systematic and narrative reviews, commentaries, and case reports encompassing molecular virology, computational epidemiology, outbreak medicine, vaccine science, maternal–fetal pathology, and public health systems science. Taken together, the manuscripts illustrate how rapidly the mpox field has matured from descriptive outbreak reporting toward an integrated and increasingly rigorous scientific discipline.

### 2.1. Viral Circulation, Genomic Evolution, and Surveillance

Several contributions advance understanding of the geographic circulation and molecular evolution of MPXV across both endemic and newly affected regions. Dorcoo and colleagues reported retrospective serological evidence indicating orthopoxvirus exposure in Ghana prior to the global clade IIb outbreak, despite the absence of previously confirmed human cases [6]. These findings reinforce the hypothesis that MPXV circulation in parts of Africa has been substantially underrecognized due to limited surveillance capacity and diagnostic infrastructure.

Shi et al. conducted phylogenetic and molecular evolutionary analyses of MPXV in Shenzhen, China, between 2023 and 2024, documenting the increasing globalization of viral dissemination and underscoring the necessity of sustained genomic surveillance well beyond traditionally endemic regions [7]. Complementary evidence emerged from case reports characterizing MPXV emergence in Mali and Kinshasa. The genomic confirmation of Mali’s index case demonstrated the feasibility of rapid molecular characterization in resource-constrained settings [8], while reports from Kinshasa documented the concurrent introduction and urban spread of both clade IIb and clade Ib viruses into one of Africa’s most densely populated metropolitan centers [9,10]. Collectively, these studies confirm that MPXV now circulates within increasingly interconnected regional and international transmission networks that resist containment through geographically limited responses.

The computational modeling study by Idisi and colleagues provided important complementary insights, employing mathematical and data-driven epidemiological frameworks to characterize differential transmission dynamics between clade I and clade II viruses and to assess the implications of cross-species interfaces for outbreak amplification [11].

### 2.2. Clinical Severity, Disease Heterogeneity, and Emerging Epidemiology

The prospective cohort investigation by Nkengurutse and colleagues from Burundi represents one of the most substantive clinical contributions of this Special Issue [12]. Their systematic analysis of hospitalized mpox patients during an active outbreak identified predictors of serious complications, reinforcing the recognition that mpox cannot uniformly be characterized as a mild self-limiting illness. These findings are particularly relevant for African outbreak settings, where delayed healthcare presentation, co-infections, malnutrition, and constrained healthcare resources may substantially amplify morbidity and mortality.

Case reports from the DRC similarly illuminate the changing clinical and epidemiological profile of current outbreaks. Historically, mpox in Central Africa disproportionately affected children in rural and forested communities. The manuscripts presented in this Issue describe a marked shift toward sexually active adults, urban populations, and mobile networks interconnected through occupational and commercial activities [9,10]. This epidemiological transition carries significant implications for transmission dynamics, prevention messaging, and the design of community-level interventions.

A recurring theme across multiple contributions is that mpox severity is not biologically uniform across populations. Disease burden appears amplified among immunocompromised persons, pregnant women, and socially marginalized groups—findings that collectively underscore the urgent need for more nuanced and stratified clinical risk assessment frameworks in both research and healthcare settings.

### 2.3. Countermeasures: Therapeutics, Antivirals, and Vaccine Science

Substantial progress in countermeasure development is reflected across multiple contributions, though these advances are consistently shadowed by concerns regarding equitable access and implementation.

In the domain of antiviral research, Alvarez-de Miranda and colleagues developed fluorescent clade IIb MPXV reporter viruses designed to facilitate high-throughput antiviral screening [13]. This methodological innovation provides an important platform for accelerating the evaluation of candidate therapeutics and expanding the countermeasure pipeline beyond currently available agents. Complementing this work, Witwit and colleagues addressed the urgent concern of potential tecovirimat resistance, which has emerged as a clinical and public health vulnerability given the current near-exclusive reliance on this compound [14]. Their exploration of synergistic repurposed drug combinations provides a compelling rationale for diversification of the antiviral therapeutic strategy and signals a critical need for investment in combination regimen research before resistance becomes widespread.

Vaccine science is also entering a period of renewed innovation following decades of relative stagnation following smallpox eradication. Williamson reviewed next-generation vaccine strategies targeting both monkeypox and capripoxviruses, identifying multiple promising platforms including modified vaccinia Ankara (MVA)-based constructs, DNA vaccines, and subunit formulations [15]. Bai and colleagues reported enhanced immunogenicity and receptor binding affinity associated with chimeric A35R-Fc protein constructs compared to native A35R protein alone, suggesting potential for improved next-generation subunit vaccine candidates [16]. These contributions collectively advance the scientific basis for MPXV vaccine development beyond first-generation products.

However, the Special Issue is equally clear that biomedical innovation in countermeasures is insufficient without equitable access. The review by Danladi and colleagues on global inequities in vaccine distribution documents in sobering detail how procurement and supply chain failures, intellectual property constraints, cold chain requirements, and affordability barriers collectively prevent life-saving vaccines from reaching the populations most severely affected by mpox outbreaks [17]. The lesson is unambiguous: scientific innovation and operational equity must advance in tandem. Neither is meaningful without the other.

### 2.4. Mpox in Pregnancy, Infants, and Children: A Critical and Underrecognized Burden

Among the most urgent themes emerging from this Special Issue is the disproportionate and severely underappreciated burden of mpox in pregnancy and among infants and children. For decades, the medical literature on MPXV largely centered on adult males; the reproductive health consequences of infection received comparatively little systematic attention.

The commentary by Schwartz provides one of the most comprehensive discussions currently available on placental pathology, maternal–fetal MPXV transmission, and strain-specific differences across clades Ia, Ib, IIa, and IIb [3]. The evidence assembled demonstrates that mpox during pregnancy may represent one of the most severe manifestations of orthopoxvirus infection currently recognized. Following the initial description by Schwartz et al. [18,19] of the placental and fetal pathology from Congenital MPOX Syndrome, recent studies have confirmed that Clade I infections, particularly in Central Africa, are associated with profoundly elevated rates of fetal demise as well as other adverse obstetrical outcomes, including missed abortions, spontaneous abortions, and stillbirth [4,20]. The mechanism proposed by Dashraath et al. [21] for maternal–fetal MPXV transmission following maternal viremia—with the virus passing through uterine blood vessels into the placenta, infecting chorionic villi, and subsequent transplacental transmission to the fetus—has been supported by identifying placental pathology including villitis, intervillositis, and viral inclusions within trophoblasts. This appears to constitute a critical pathological mechanism underlying adverse fetal outcomes. Strain-specific differences in placental tropism and pathogenicity are emerging as a scientifically important and clinically urgent area of investigation.

The review by Imran and colleagues synthesizes available evidence on vaccination and antiviral therapies during pregnancy and breastfeeding, identifying significant gaps in the evidence base [22]. Current regulatory frameworks largely exclude pregnant women from clinical trials on ethical grounds, yet this exclusion itself generates a profound and dangerous evidence vacuum—one that leaves clinicians without guidance precisely when the stakes for both mother and fetus are highest. The authors emphasize the need for prospective pregnancy registries, compassionate-use frameworks for antiviral access, and vaccine safety monitoring systems capable of capturing maternal and neonatal outcomes.

Historically, children represented the primary demographic affected by mpox in endemic African settings. The recent epidemiological shift toward adults—particularly in the context of sexual transmission networks—should not obscure the continued vulnerability of pediatric populations, especially in communities where household transmission remains a dominant route. The contributions in this Special Issue highlight that children in endemic settings remain substantially underrepresented in clinical research, diagnostic validation studies, and therapeutic trials. Dedicated pediatric investigation is a scientific and ethical imperative.

### 2.5. Mpox Among People Living with HIV and Immunocompromised Populations

The review by Orkin and colleagues examining mpox among people living with HIV (PLHIV) reinforces the clinical significance of intersecting epidemics and the necessity of integrated programmatic responses [23]. Among individuals with advanced immunosuppression—particularly those with low CD4 counts and unsuppressed HIV viral loads—mpox may present with atypical features, prolonged viral shedding, progressive or disseminated disease, and substantially elevated mortality. The review documents that vaccination coverage among PLHIV remains unacceptably low in many affected settings, despite clear evidence of benefit, and that therapeutic access is similarly inequitable.

These findings argue compellingly for the co-integration of mpox screening, prevention, and treatment into existing HIV service delivery platforms—a structural approach that could simultaneously improve access, reduce stigma, and leverage existing community trust networks.

### 2.6. Community Engagement, Stigma, and Implementation Science

Although no single manuscript in this Special Issue was exclusively dedicated to community engagement as a primary focus, the theme emerges with consistent urgency across multiple contributions. The 2022 global outbreak demonstrated both the indispensable value of community-led responses—particularly among GBMSM communities who rapidly mobilized peer outreach, harm reduction, and vaccine advocacy efforts—and the destructive consequences of disease-associated stigma, which delayed healthcare seeking, complicated contact tracing, and undermined trust in public health authorities.

The commentary by Gashema and colleagues, drawing lessons from COVID-19 and Ebola response experiences in East Africa, explicitly underscores that biomedical countermeasures without a robust community engagement infrastructure will consistently underperform their potential [24]. Community health workers, peer navigators, trusted civil society organizations, and traditional leaders represent essential—not supplemental—components of effective outbreak response, particularly in settings where formal healthcare systems are under-resourced or historically regarded with distrust.

Wayengera and colleagues’ commentary on restoring orthopoxvirus herd immunity contributes a population-level dimension to this Special Issue, arguing that sustainable mpox control requires not only reactive outbreak response but proactive and inclusive vaccination strategies that rebuild the immunity gap created by smallpox vaccine discontinuation [25].

## 3. Major Conceptual Shifts Emerging from This Special Issue

### 3.1. From Zoonotic Spillover to Sustained Human Pathogens

Perhaps the most significant conceptual reorientation arising from this Special Issue is the recognition that mpox should no longer be conceptualized primarily as a sporadic zoonotic disease. The evidence assembled across these nineteen manuscripts demonstrates increasingly efficient MPXV human-to-human transmission across multiple epidemiological contexts—sexual networks, household contacts, healthcare settings, and birth transmission.

The coexistence of sexual transmission amplification, urban household spread, and ongoing zoonotic interfaces indicates that MPXV appears to occupy a far more complex ecological niche than previously recognized. This complexity substantially complicates both outbreak containment strategies and long-term control frameworks, and suggests that future transmission patterns will remain heterogeneous, context-dependent, and difficult to predict based on historical analogy.

### 3.2. Africa as the Epicenter of Scientific and Public Health Importance

Although the 2022 outbreak internationalized scientific and public attention toward mpox, the most consequential epidemiological, biological, and clinical developments continue to emerge from African settings. The DRC, Burundi, and neighboring countries are generating the world’s most important evidence regarding clade evolution, severe disease manifestations, maternal–fetal outcomes, pediatric burden, and transmission dynamics.

However, these same settings remain among the least resourced in terms of laboratory capacity, vaccine availability, antiviral access, genomic sequencing infrastructure, and clinical research capacity. This contradiction—the world’s most scientifically important mpox burden being concentrated in the world’s most neglected research environments—represents one of the most troubling structural failures exposed by this Special Issue. The future trajectory of mpox will depend heavily on developments in endemic African settings. Sustainable, locally led investment in surveillance, diagnostics, genomics, and community-embedded clinical research is therefore not merely an equity obligation—it is a global preparedness imperative.

### 3.3. The Equity Gap Between Biomedical Innovation and Operational Deployment

A third major conceptual shift concerns the widening divergence between scientific advancement and equitable implementation. The field is progressing in genomic sequencing, computational modeling, immunogenic target discovery, antiviral development, and vaccine innovation. However, endemic countries continue to face critical shortages of diagnostics, delays in vaccine procurement, weak laboratory systems, absent or fragmented surveillance, and systematic exclusion from the therapeutic clinical trials generating the evidence that ultimately guides global treatment guidelines. This asymmetry mirrors patterns repeatedly observed across infectious disease emergencies—such as HIV, Ebola, and COVID-19—and its structural determinants, including the consequences of donor funding contraction and the post-PHEIC transition to endemic disease management, are examined in the accompanying Policy Review published in this Issue.

## 4. Persistent Gaps and Unresolved Research Priorities

Despite the substantial progress documented in this Special Issue, several critical deficiencies remain apparent. Longitudinal clinical data on long-term sequelae, post-infection immunity, and reproductive outcomes are almost entirely absent. Pregnant women, breastfeeding mothers, infants, and children remain systematically excluded from vaccine and antiviral trials, producing an evidence vacuum with directly harmful clinical consequences. Implementation science—covering vaccine hesitancy, stigma reduction, community engagement, and health-seeking behavior in endemic settings—is critically underdeveloped. Further, global preparedness structures remain fundamentally reactive, mobilizing after transcontinental spread rather than during endemic amplification, when intervention would be more feasible and cost-effective. The programmatic and institutional responses required to address each of these gaps—including the transition to routine care, integration into HIV and STI platforms, and the roles of national and continental public health bodies in sustaining surveillance and research—are examined in detail in the accompanying Policy Review published in this Special Issue.

## 5. Future Directions

Translating the scientific advances documented in this Special Issue into sustained, equitable, and institutionally grounded public health practice is the defining challenge of the post-emergency mpox landscape. The closure of the WHO PHEIC in 2023—followed by its reissuance in 2024 in response to clade Ib escalation—exposed the structural fragility of emergency-dependent response architectures and the urgency of transitioning mpox into routine health system management. This transition requires integrated surveillance platforms, domesticated program financing, inclusive clinical research, and clearly defined roles for national and continental public health institutions operating under conditions of fiscal constraint. A detailed programmatic framework for this transition—encompassing the integration of mpox into HIV and STI services, the mandates of National Institutes of Public Health, EPI programs, national reference laboratories, Africa CDC, and WHO, and a forward-looking research agenda—is developed in the accompanying Policy Review published in this Special Issue: Katoto et al., “After the Emergency: Sustaining Mpox Surveillance, Research, and Care Integration in a Post-PHEIC, Resource-Constrained World”.

## 6. Conclusions

This Special Issue concludes at a critical juncture in the evolution of mpox research. The nineteen manuscripts assembled here collectively document that MPXV is no longer a peripheral or neglected pathogen but a dynamic, evolving, and multidimensional global health challenge requiring sustained multidisciplinary scientific engagement and long-term international commitment.

These contributions substantially advance scientific understanding of viral evolution and geographic spread, clinical severity and heterogeneity, therapeutic innovation, vaccine science, maternal–fetal disease, pediatric burden, HIV-associated outcomes, community dynamics, and outbreak preparedness. They also, with equal clarity, reveal persistent and deepening weaknesses in surveillance infrastructure, implementation capacity, equity of access, and the governance frameworks that ultimately determine whether scientific advances translate into population health benefits.

The burden of mpox in pregnancy and among infants and children commands particular and urgent attention. The evidence assembled in this Special Issue indicates that these groups face some of the most severe manifestations of MPXV infection, yet remain the most systematically excluded from the research that could guide their care and protection. Progress in countermeasures—antivirals, vaccines, and diagnostics—has been real and meaningful. Whether that progress reaches the communities most in need of it will depend not on further scientific breakthroughs alone, but on the political will, governance structures, and sustained investment required to translate innovation into equity. Mpox now stands as both a biological challenge and a test of global health solidarity. Research developments are advancing; the remaining question—as it has been across so many previous infectious disease emergencies—is whether scientific progress will be translated into sustainable and equitable implementation.

## Data Availability

No new data were created or analyzed in this study. Data sharing is not applicable to this article.
